# A rare cause of small intestinal obstruction in children: a case report of calcifying fibrous tumor

**DOI:** 10.1186/s12887-025-06149-8

**Published:** 2025-09-19

**Authors:** Zesheng Yang, Xiaoying Xie, Shicheng Wang, Weijun Xu, Guanghua Pei, Jun Wan

**Affiliations:** 1 Department of Ultrasound, Tianjin Children’s Hospital (Children’s Hospital of Tianjin University), No. 238 Longyan Road, Beichen District, Tianjin, 300134 China; 2 Department of Emergency and Traumatic Surgery, Tianjin Children’s Hospital(Children’s Hospital of Tianjin University), Tianjin, China

**Keywords:** Calcifying fibrous tumor, Child, Intestinal obstruction, Small intestine, Ultrasonography

## Abstract

**Background:**

Calcifying fibrous tumor (CFT) is a rare benign mesenchymal tumor. Primary small intestinal CFT causing intestinal obstruction in children is exceptionally rare. Preoperative diagnosis remains challenging due to its nonspecific clinical manifestations. Ultrasound holds potential advantages in pediatric abdominal imaging, though further studies are needed to validate its utility for rare entities like CFT.

**Case presentation:**

A 3-year-old girl presented with progressive abdominal distension for 2 months, accompanied by occasional periumbilical restlessness (suggestive of mild discomfort). No bilious vomiting was noted, and bowel movements remained regular with normal consistency. High-frequency abdominal ultrasound revealed dilated small bowel loops, focal jejunal wall thickening (6 mm) with calcified foci and posterior acoustic shadowing, and punctate blood flow signals on color Doppler imaging, collectively indicating mechanical obstruction secondary to localized calcified wall thickening. CT confirmed small bowel dilation and high-density calcified lesions. Laparoscopy-confirmed obstructive jejunal stenosis further prompted mini-laparotomy with curative jejunal resection and primary anastomosis. Histopathology and immunohistochemistry confirmed CFT diagnosis. The patient recovered uneventfully and showed no recurrence at 2-month follow-up.

**Conclusions:**

Small intestinal CFT is a rare cause of pediatric intestinal obstruction. High-frequency ultrasound demonstrated diagnostic capability in this specific case by identifying the pathognomonic triad of bowel wall thickening with calcifications and obstructive transition zone, facilitating timely preoperative assessment as a radiation-free initial imaging tool, with CT providing complementary confirmation of calcified lesions. Complete surgical resection achieved symptom resolution with no recurrence observed during available follow-up (2 months). This case supports considering small intestinal CFT in the differential diagnosis of children with unexplained chronic intestinal obstruction when the aforementioned pathognomonic triad (bowel wall thickening, calcifications, and obstructive transition zone) is observed, though larger pediatric cohort studies are required to validate this diagnostic approach.

## Introduction

Calcifying fibrous tumor (CFT) is a rare benign mesenchymal tumor characterized by hyalinized collagenous stroma containing dystrophic calcifications or psammoma bodies [1]. Initially described by Rosenthal et al. (1988) as “childhood fibroma with psammoma bodies” [2], the World Health Organization (WHO) formally classified this entity as a fibroblastic/myofibroblastic tumor in the 2002 WHO classification of soft tissue tumors, designating it “Calcifying Fibrous Tumor” (CFT) [3]. CFT can arise in diverse anatomical sites—including the pleura, gastrointestinal tract (e.g., mesentery, stomach), and subcutaneous soft tissues [4, 5], though primary small intestinal CFT is exceptionally rare in children, with only a handful of well-documented pediatric cases reported globally [6, 7]. The non-specific clinical manifestations (e.g., abdominal pain, distension) and absence of pathognomonic imaging features present significant diagnostic challenges for pediatric small intestinal CFT. This report details how high-frequency ultrasound identified characteristic sonographic features of CFT-induced jejunal obstruction in a 3-year-old child—specifically a pathognomonic triad of bowel wall thickening, calcifications, and an obstructive transition zone—highlighting its preoperative diagnostic utility in this specific clinical scenario.

## Case description

A 3-year-old girl was admitted due to progressive abdominal distension for 2 months. Clinical history revealed occasional periumbilical restlessness (e.g., frowning, transient squirming) during the course, without bilious vomiting, hematochezia, or melena. Bowel movements were documented as regular with normal consistency, and urinary function was unremarkable.

Physical examination revealed abdominal tympany and diffuse distension without visible intestinal loops, tenderness, rebound tenderness, guarding, or abnormal bowel sounds. All routine laboratory values were within normal limits.

**Fig. 1:**
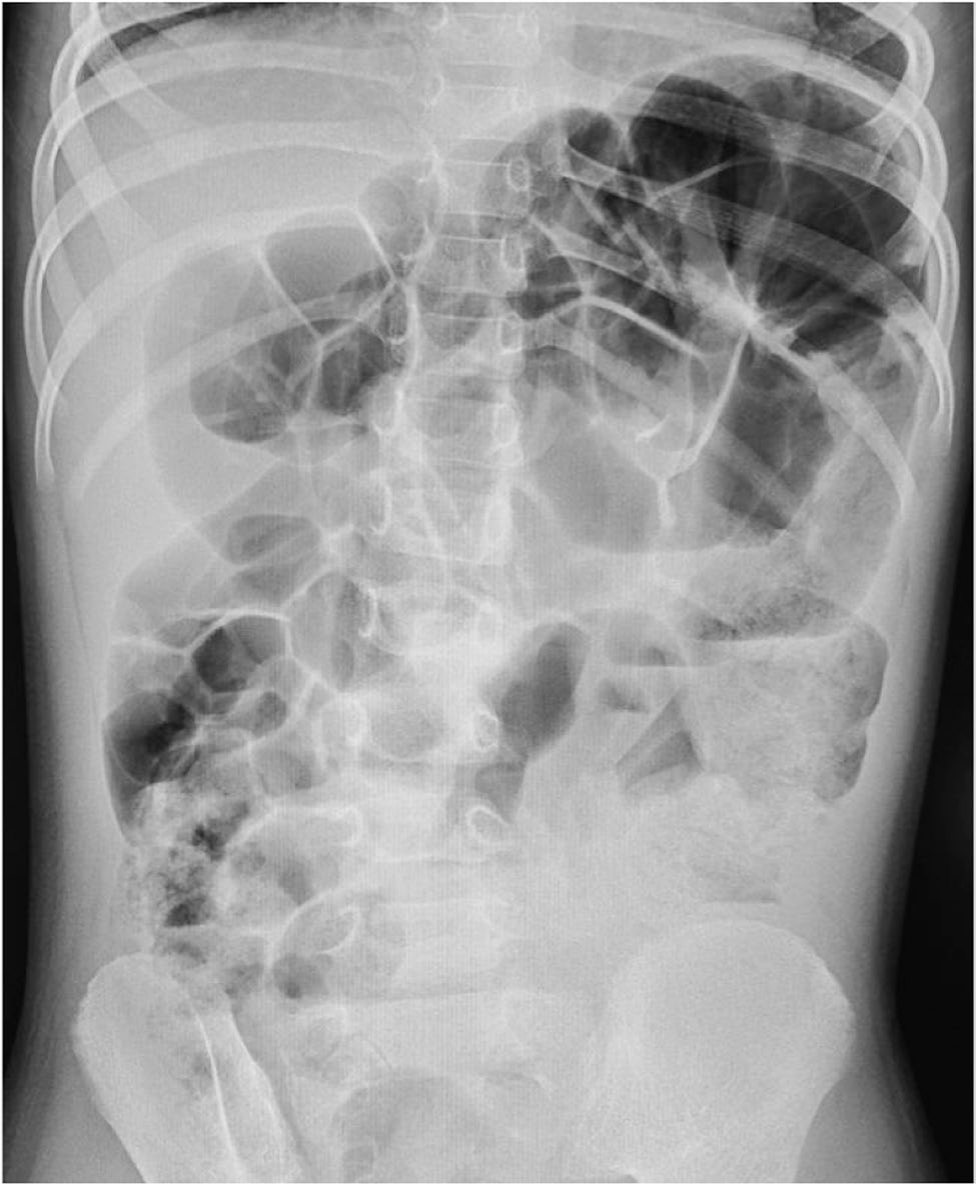
Plain erect abdominal radiograph showed diffuse intestinal gas accumulation

**Fig. 2:**
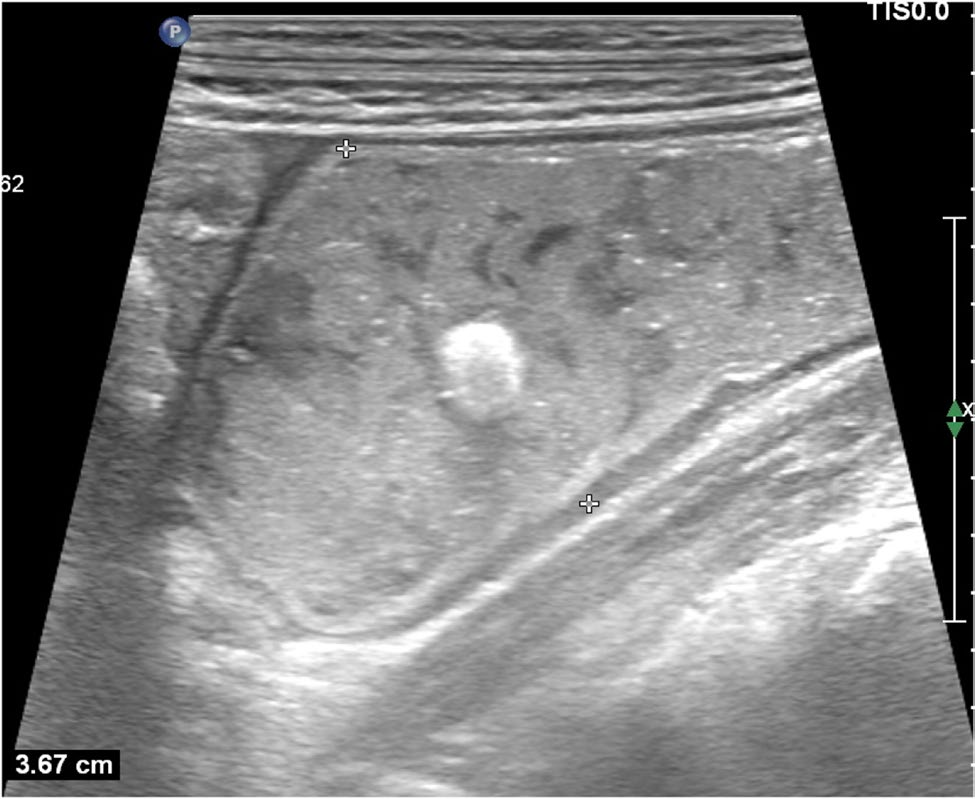
High-frequency ultrasound shows the dilated small intestine with an inner diameter of 3.7 cm

**Fig. 3:**
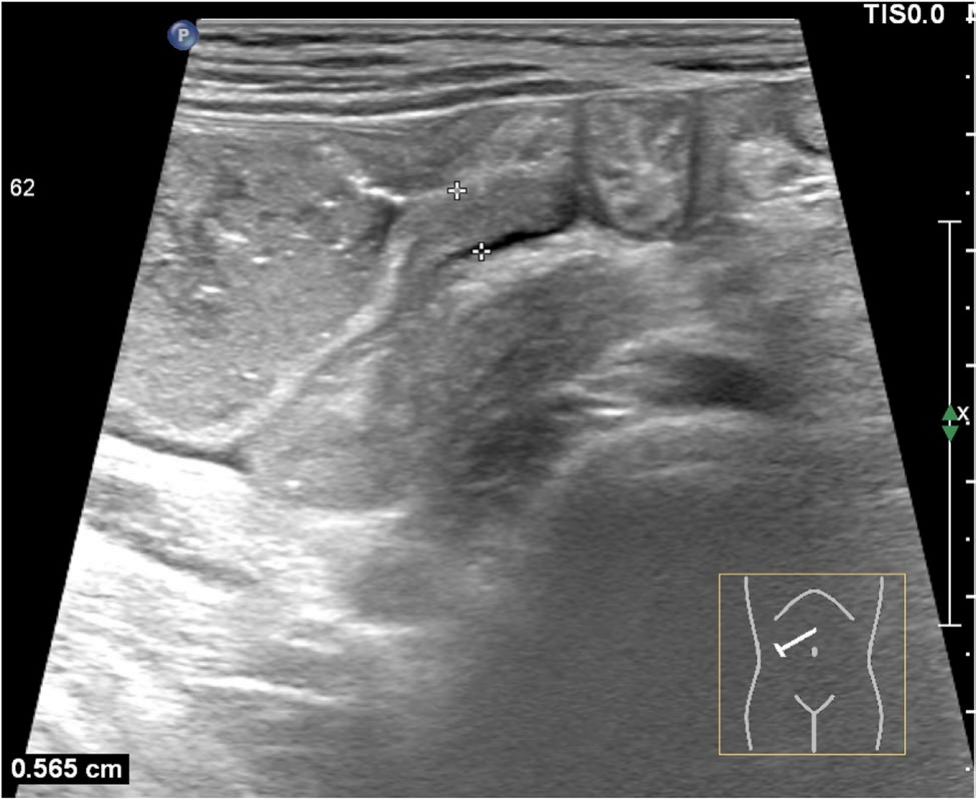
The intestinal wall of the jejunum stenosis segment is thickened by 6 mm, presenting as hypoechoic

**Fig. 4:**
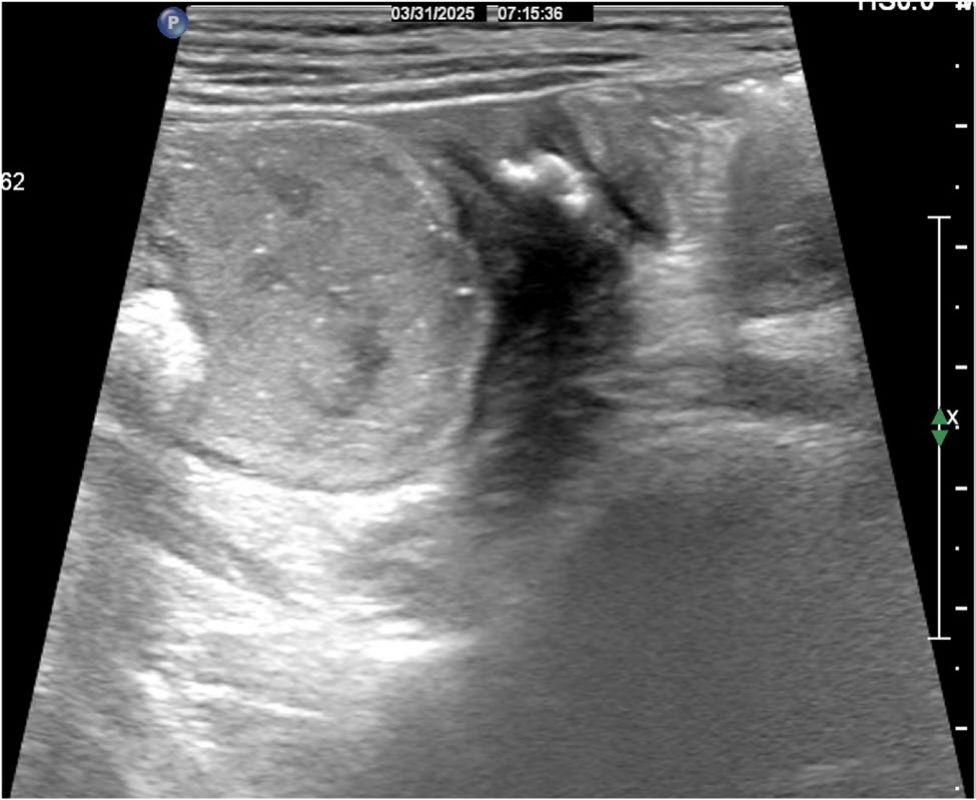
Several calcification spots with an acoustic shadow are seen within the thickened intestinal wall

**Fig. 5:**
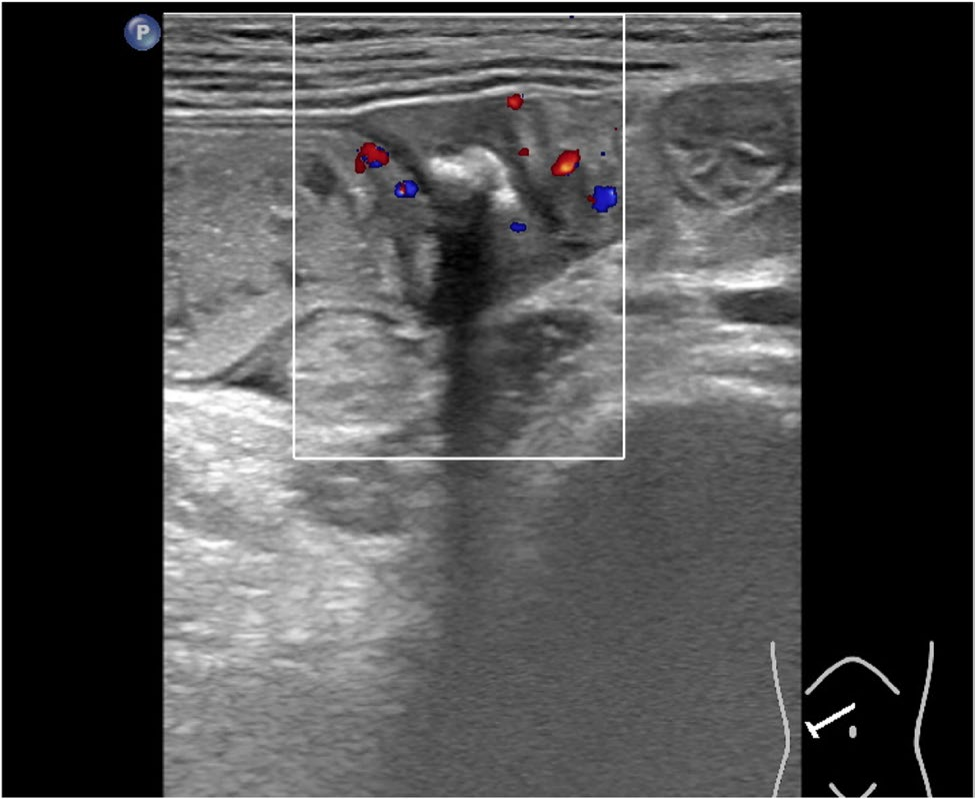
CDFI shows punctate blood flow signals in the lesion

**Fig. 6:**
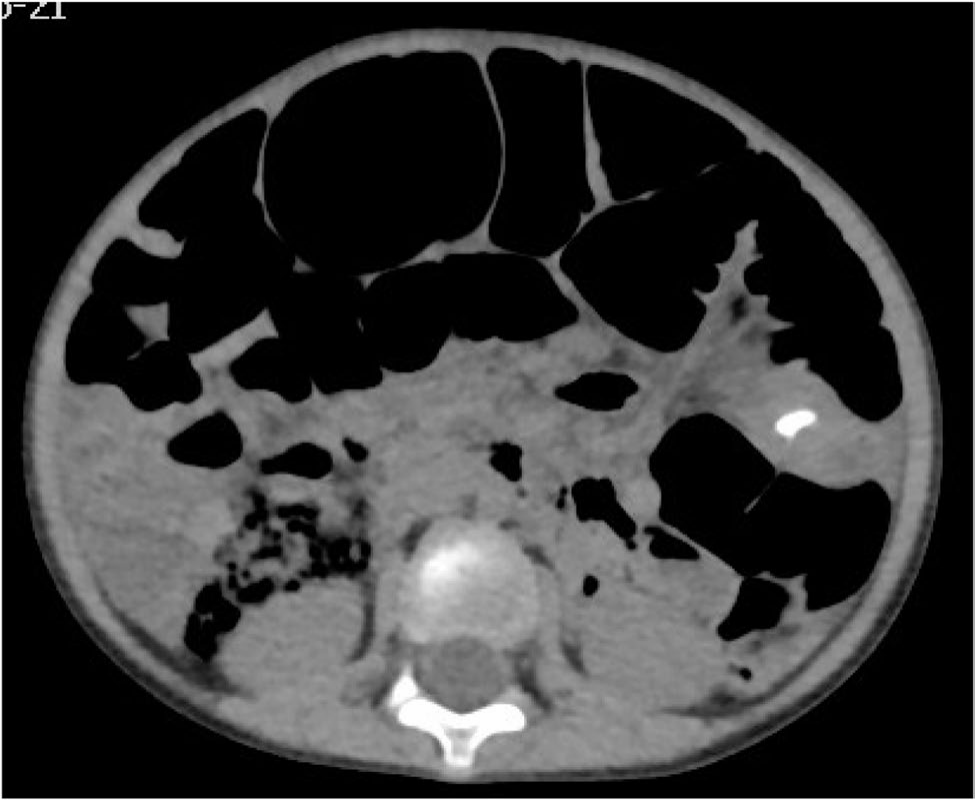
CT plain scan shows a high-density calcification shadow in the left mid-abdomen

Plain erect abdominal radiograph showed diffuse intestinal gas accumulation with no free air under the diaphragm, supporting the initial suspicion of intestinal obstruction (Fig. 1). High-frequency abdominal ultrasound (5–12 MHz) revealed markedly dilated small bowel loops (Fig. 2), and key features of a pathognomonic triad: a transition zone at the umbilical region (obstructive transition zone), focal bowel wall thickening (6 mm), and hyperechoic calcifications with acoustic shadowing in the thickened wall (Figs. 3 and 4). Punctate vascularity was noted on color Doppler imaging (Fig. 5). These findings collectively indicated mechanical obstruction secondary to localized calcified bowel wall thickening. Abdominal CT further confirmed dilated small bowel loops with air-fluid levels and high-density calcific foci in the left mid-abdomen (Fig. 6).

After 72 h of conservative management (nasogastric decompression and intravenous fluid resuscitation), persistent abdominal distension prompted repeated ultrasonography. This revealed persistent small bowel dilation and a fixed transition zone, confirming mechanical obstruction. Given refractory symptoms and high risk of bowel compromise (ischemia/perforation), laparoscopic exploration was therefore indicated.

Preoperative differential diagnoses, based on clinical and imaging findings, included but were not limited to:

- Intussusception: Ruled out due to absence of paroxysmal abdominal pain and lack of the “concentric circle sign” on ultrasound.
- Congenital intestinal stenosis: Unlikely given the presence of calcifications—a feature rare in congenital lesions.
- Gastrointestinal stromal tumor (GIST): Considered improbable owing to its pediatric rarity, absence of hypervascular features, and infrequency of calcifications in GIST.
- Inflammatory myofibroblastic tumor: Less probable because calcifications are uncommon in such tumors.
- Lymphoma: Excluded as neither ultrasound nor CT demonstrated diffuse bowel wall thickening (“pseudokidney sign”) or associated lymphadenopathy.

**Fig. 7:**
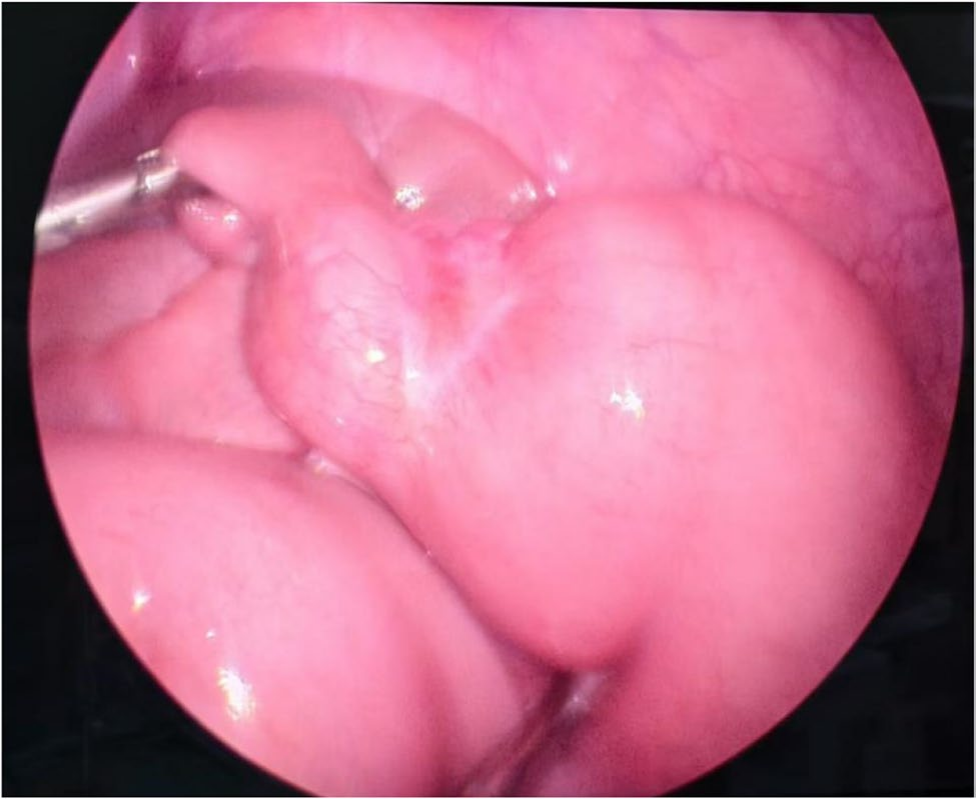
Intraoperative view shows the thick-thin junction segment of the jejunum

Laparoscopic exploration revealed markedly dilated jejunum with hyperperistalsis and retrograde peristaltic waves. A distinct transition zone was identified at 200 cm proximal to the ileocecal valve and 120 cm distal to the ligament of Treitz (Fig. 7), characterized by proximal dilation, distal collapse, and an intervening rigid stenotic segment; complete abdominal survey demonstrated no abnormalities. Following extension of the umbilical incision to 5 cm, exteriorization revealed: proximal bowel dilation (5 cm diameter) with wall thickening (6–8 mm), distal collapse (1.5 cm diameter) without wall thickening, and complete luminal occlusion at the rigid stenotic segment preventing content passage, confirming mechanical obstruction. En bloc resection of the diseased segment was performed; given significant luminal disparity (5 cm vs. 1.5 cm), proximal tapering via 4-cm antimesenteric wedge resection achieved caliber matching, followed by end-to-end anastomosis with anastomotic patency confirmed by air insufflation test.

**Fig. 8:**
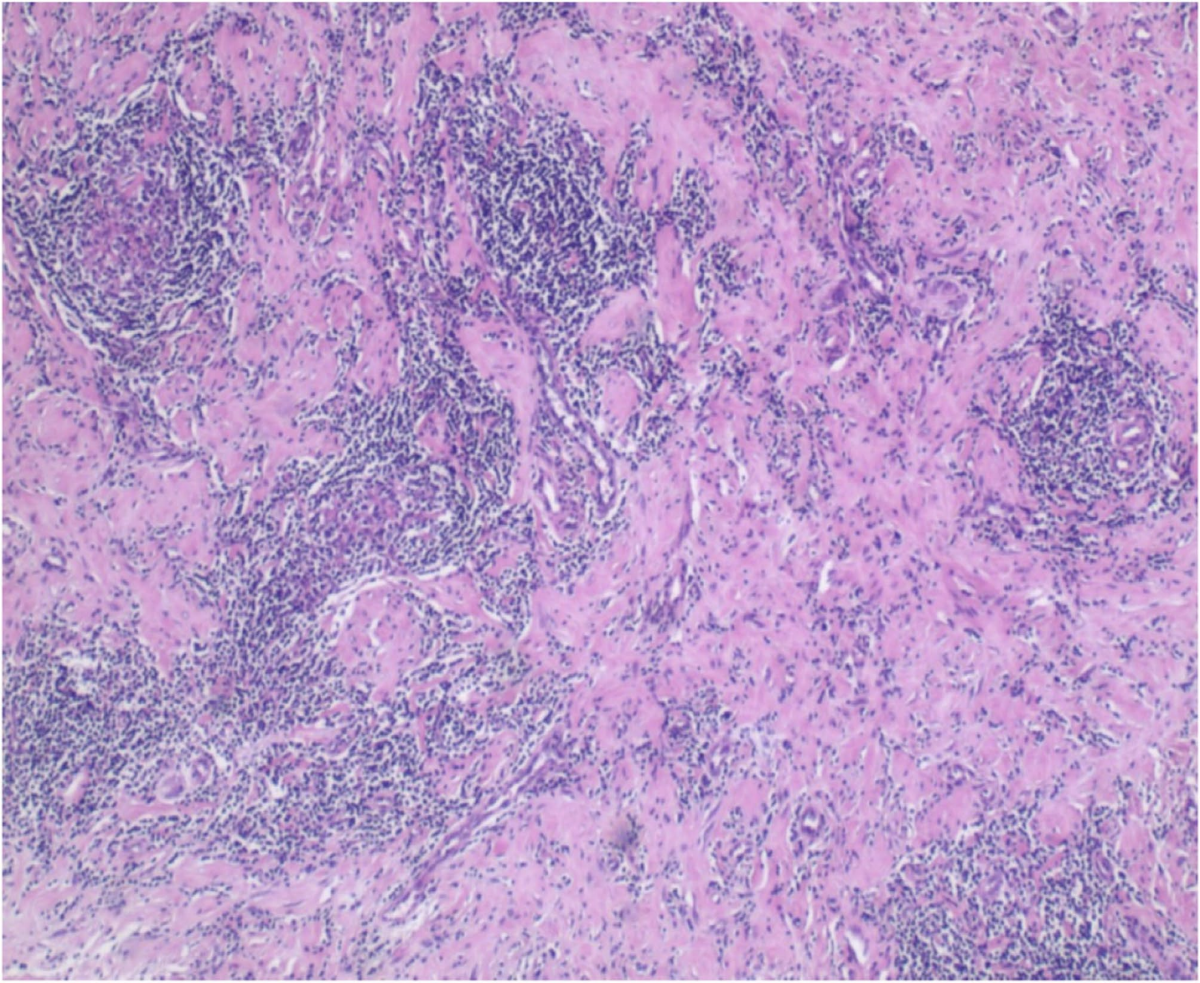
Pathological HE staining shows fibroblastic hyperplasia with hyaline degeneration (×40)

**Fig. 9:**
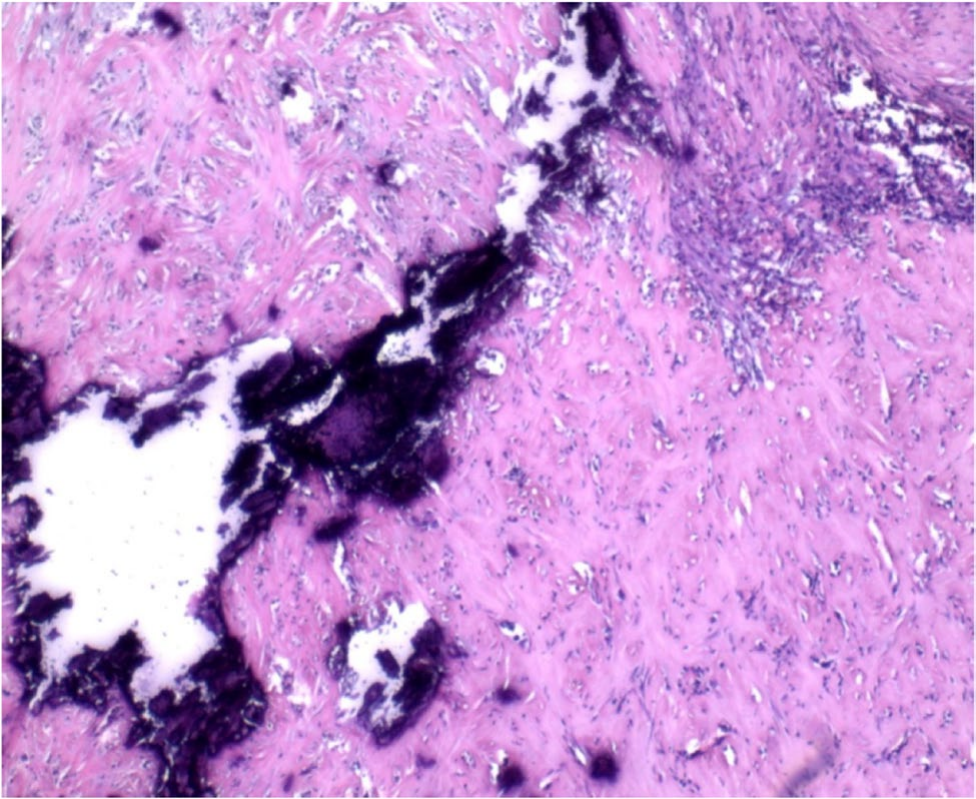
Calcium salt deposition in the serosa layer (×40)

Pathology confirmed calcifying fibrous tumor (CFT): Histology showed mucosal/submucosal fibroblastic/myofibroblastic proliferation, hyalinized collagen deposition with lymphocytic infiltration (Fig. 8), and serosal dystrophic calcifications (Fig. 9). IHC demonstrated focal SMA/desmin/caldesmon positivity, ALK/CD34/CD117 negativity, and Ki-67 index of 3%.

The patient was discharged on postoperative day 8, demonstrating uncomplicated recovery. At the 2-month follow-up, she remained asymptomatic without clinical or ultrasonographic evidence of recurrence. However, this short follow-up period limits assessment of long-term recurrence risk, and extended surveillance is planned.

## Discussion

The pathogenic mechanisms underlying CFT remain incompletely elucidated. Current theories posit CFT to be a reactive fibrosing process triggered by chronic inflammation or tissue injury [8], though validation requires larger cohort studies. Within the gastrointestinal tract, mucosal microtrauma due to mechanical food passage is proposed as a potential contributor. This pediatric case illustrates the clinical relevance: protracted abdominal distension (spanning 2 months) suggests chronic luminal content stasis potentially exacerbating localized tissue damage. Notably, this mechanistic pathway currently lacks direct molecular evidence establishing causal relationships.

Chorti et al.‘s systematic review (*n* = 157) reported a mean age of 33.58 years (range: 5 weeks–84 years). Pediatric cases constituted 8.3% (13/157), with fewer than 5 primary small intestinal CFTs [1]. Bimodal age peaks occurred predominantly at 0–4, 25–29, and 30–34 years, with female predominance (male-to-female ratio: 1:1.27) [1]. This case—a 3-year-old girl with jejunal involvement aligns with pediatric epidemiological patterns.

CFT typically exhibits occult growth with nonspecific manifestations. Asymptomatic cases are often incidental findings; symptomatic patients may present with: digestive complaints (e.g., abdominal pain, anorexia) or systemic symptoms (e.g., fatigue, weight loss) [8]. Severe complications include obstruction, intussusception, or volvulus [8, 9]. The clinical heterogeneity of CFT is underscored by contrasting reports: Kim et al. documented mesenteric CFT masked by traumatic bowel perforation [10], exemplifying diagnostic challenges; conversely, Sotiriou et al. established small intestinal CFT as a validated rare etiology for intussusception [11], highlighting its potential to mimic common abdominal emergencies.

Notably, CFT may demonstrate relatively distinctive imaging features on ultrasonography, including well-defined hypoechoic masses with internal calcifications and posterior acoustic shadowing—these findings that align with established pediatric mesenteric CFT characteristics [5]. In this pediatric case, high-frequency ultrasound identified focal jejunal wall thickening (6 mm) with discrete calcifications, and dynamically visualized the transition zone between dilated and collapsed bowel segments, providing direct evidence of mechanical obstruction via real-time peristaltic arrest documentation.

Non-contrast CT further demonstrated soft-tissue density lesions containing punctate or clustered high-density calcifications, precisely delineating anatomical relationships with adjacent mesenteric vessels. While ultrasound excels in real-time assessment of bowel dynamics, CT provided critical complementary information through:

1. Exclusion of multifocal involvement (multifocal lesions reported in 9 of 157 CFT cases [5.73%] [1]);
2. Characterization of calcification distribution patterns;
3. Morphological discrimination between calcification types (e.g., irregular dystrophic calcifications versus homogeneous osteoid deposits).

Crucially, while these features are highly suggestive, definitive diagnosis of CFT requires histopathological and immunohistochemical confirmation [12].

Surgical resection constitutes the definitive management for CFT, with its characteristic benign biological behavior supported by established literature [1, 13]. The child showed no signs of recurrence during the initial 2-month follow-up period, though long-term surveillance remains essential to confirm durable remission. Continued monitoring beyond this timeframe is warranted.

This study has several limitations. First, it is a single-case report, so the clinical and imaging features described may not fully represent the spectrum of pediatric small intestinal CFT. Second, the follow-up period was only 2 months, which limits our ability to evaluate long-term recurrence risk and the durability of surgical outcomes. Future multi-center studies with larger cohorts and longer follow-up are needed to better characterize the disease, validate the diagnostic value of ultrasound, and optimize management strategies.

## Conclusion

Small intestinal CFT is a rare cause of intestinal obstruction in children, with diagnosis relying on recognition of its characteristic triad: bowel wall thickening, calcifications, and an obstructive transition zone. High-frequency abdominal ultrasound, as a radiation-free initial imaging tool, plays a valuable role in identifying these pathognomonic features, aiding timely preoperative assessment in pediatric patients. This case underscores the need to include CFT in the differential diagnosis of chronic pediatric intestinal obstruction when the aforementioned triad is observed. Further studies with larger cohorts are warranted to validate these findings.

## Data Availability

All clinical data, imaging files, and pathological results generated or analyzed during this study are included in this published article and its supplementary information files (including ultrasound images and pathology slides). Patient identifiers have been removed to protect confidentiality.
